# Supplementary material for: An aspartyl protease defines a novel pathway for export of Toxoplasma proteins into the host cell
Source: eLife. 2015 Nov 18;4:e10809. doi: 10.7554/eLife.10809 (PMC4764566; doi:10.7554/eLife.10809)
Supplement: Supplementary file 1. — DOI: http://dx.doi.org/10.7554/eLife.10809.021 [file elife-10809-supp1.docx]

***Supplementary File 1: Primers used in this study*.**

| # | Primer Sequence |
| --- | --- |
| 1 | CGAGGTCGACGGTATCGATAATGTTCCTGTTCCAGACAG |
| 2 | TACATAGATCGACGGTATCCGCACAAAG |
| 3 | CGAAGTTATACTAACCAGAAGGAGGTGAC |
| 4 | CGCTCTAGAACTAGCTAGTGATCTACCGTTCTGCTCATG |
| 5 | GGATACCGTCGATCTATGTATCGAAACCACTC |
| 6 | GGCCTCGGACCGCTAGGCATAATCTGGAAC |
| 7 | ATGCCTAGCGGTCCGAGGCCTGACTACG |
| 8 | TTCTGGTTAGTATAACTTCGTATAATGTATGCTATACGAAGTTATG |
| 9 | CACCTGACGGATTAGTCAGTGCGGCCGCTGCTGAGGAACCTC |
| 10 | GAGGTTCCTCAGCAGCGGCCGCACTGACTAATCCGTCAGGTG |
| 11 | AGTCGGCCGGCCCTTAAGCTGCCAAAGCCATGCCTC |
| 12 | AGTCATGCATACATAAGCGCGTACATG |
| 13 | AGTCAGATCTTTAGCCATATGGAGGC |
| 14 | AGTCCCCGGGCTTAAGAGACCTGAGCACGCG |
| 15 | TCTCCTCAACGTTTTAGAGCTAGAAATAGCAAG |
| 16 | CGGGGACGGACAACTTGACATCCCCATTTAC |
| 17 | CTGGGCAGATGTTTTAGAGCTAGAAATAGCAAG |
| 18 | AACAGGACCCAACTTGACATCCCCATTTAC |
| 19 | GGTGTCGTATGGACTCTCCGTGTGG |
| 20 | GCTGCACTGCTTGCGGACGCAGATTCAAAGAGG |
| 21 | GGGGGCTCTTCGTTCCTCAGTTTCTCC |
| 22 | CCTGTGCCCGCTTACGGCGTCTCATACCATGTTTTCG |
| 23 | GGAACTGACACAGCCTCCGAGAG |
